# AngioJet rheolytic thrombectomy under superior vena cava filter protection for acute upper extremity deep vein thrombosis in patients with cancer: a single-center, retrospective analysis

**DOI:** 10.1016/j.rpth.2026.107002

**Published:** 2026-10-01

**Authors:** Shaobo Zhai, Jia Yu, Jifeng Sun, Yangyang Yu, Cheng Gao

**Affiliations:** 1 Department of Vascular Surgery, Qingdao Central Hospital, University of Health and Rehabilitation Sciences, Qingdao, People's Republic of China; 2 Department of Geriatrics, Qingdao Central Hospital, University of Health and Rehabilitation Sciences, Qingdao, People's Republic of China

**Keywords:** neoplasms, pulmonary embolism, thrombectomy, upper extremity deep vein thrombosis, vena cava filters

## Abstract

**Objective:**

The incidence of upper extremity deep vein thrombosis (UEDVT) is increasing, and percutaneous mechanical thrombectomy (PMT) is receiving more attention as a treatment option. This study aims to evaluate the efficacy and clinical outcomes of PMT combined with superior vena cava (SVC) filter protection in cancer patients with acute UEDVT.

**Methods:**

This study included all patients with acute UEDVT who underwent treatment with an 8-Fr AngioJet thrombectomy system (Boston Scientific) under the protection of a retrievable SVC filter between January 1, 2020, and October 1, 2025. The evaluation indicators included technical success rate (defined as a thrombus clearance rate >80%), intraoperative adverse events, degree of limb swelling relief, incidence of pulmonary embolism, and thrombus recurrence during follow-up.

**Results:**

A total of 19 patients (10 female; mean age 60.84 ± 11.30 years) were included. All patients had secondary UEDVT and a history of malignant tumors, with 14 cases (73.7%) having a history of central venous catheterization. All procedures achieved technical success, with no severe adverse events such as major bleeding occurring intraoperatively, nor were there any new symptomatic pulmonary embolisms. The average circumference of the affected limb 10 cm above the elbow before surgery was 29.83 ± 2.20 cm, which decreased to 26.98 ± 2.0 cm on the second postoperative day. During the 6-month follow-up, one patient with advanced lung cancer developed swelling of the upper limb on postoperative day 136, confirmed by ultrasound to be a recurrence of deep vein thrombosis; another patient was found to have an asymptomatic mural thrombus on ultrasound examination on postoperative day 28.

**Conclusion:**

PMT with the 8-Fr AngioJet system and SVC filter protection appears to be a safe and effective treatment option for cancer patients with acute UEDVT.

## Essentials

• PMT with an 8-Fr AngioJet system under retrievable SVC filter protection was evaluated in 19 cancer patients with acute UEDVT.
• All procedures achieved technical success (thrombus clearance >80%), with no major bleeding or new symptomatic pulmonary embolism.
• Upper-limb circumference 10 cm above the elbow decreased from 29.83 ± 2.20 cm to 26.98 ± 2.0 cm by postoperative day 2.
• Thrombus recurrence occurred in 2 patients during 6-month follow-up, but the combined strategy appears safe and effective in this population.

## 1 Introduction

Acute upper extremity deep vein thrombosis (UEDVT) is a relatively rare but significantly harmful vascular disease, with typical symptoms including swelling, pain, and functional limitations of the upper limb. In severe cases, UEDVT can lead to life-threatening complications such as pulmonary embolism (PE), significantly impacting patients’ quality of life and survival prognosis [1]. With the widespread use of medical devices such as central venous catheters, the incidence of UEDVT has shown an upward trend and has become an important focus in clinical practice [[2], [3]]. Moreover, patients with malignant tumors have a significantly increased risk of UEDVT, with an estimated incidence of approximately 10%, further exacerbating the medical burden and patient suffering [[4], [5]]. Therefore, exploring effective strategies for the diagnosis and treatment of UEDVT is of significant clinical importance for reducing the risk of related complications and improving patient prognosis.

Current treatment options mainly include anticoagulation therapy, thrombolysis, and mechanical thrombectomy. Central venous catheters, particularly those with larger diameters, can induce mechanical endothelial injury and, together with venous stasis and cancer-related hypercoagulability, promote thrombus formation [6]. These changes not only favor thrombosis but may also limit the efficacy of systemic anticoagulation alone. Interventional treatment, such as percutaneous mechanical thrombectomy (PMT), has shown good thrombus clearance effects in the treatment of lower limb deep vein thrombosis [[7], [8]]. However, there are still limited clinical data regarding the application of PMT in acute UEDVT. In addition, because of the unique anatomical structure and hemodynamic characteristics of upper extremity vessels, there is a potential risk of thrombus dislodgement leading to PE during mechanical thrombectomy [9].

In response to the above challenges, the application of PMT combined with superior vena cava (SVC) filter protection has gradually gained attention. The SVC filter can effectively intercept dislodged thrombi to prevent them from entering the pulmonary circulation, thereby reducing the risk of PE [[10], [11]]. However, there are still few reports on the clinical efficacy and safety of using mechanical thrombectomy systems (especially the AngioJet system; Boston Scientific) for the treatment of acute UEDVT under filter protection. This study aims to evaluate the technical success rate, safety, and short-term clinical outcomes of using AngioJet mechanical thrombectomy for the treatment of patients with acute UEDVT under SVC filter protection.

## 2 Methods

### 2.1 Study design and ethical approval

This study is a single-center, retrospective analysis aimed at evaluating the clinical outcomes of patients with acute UEDVT who received endovascular treatment. All included patients underwent PMT. Ethical approval was granted by the Ethics Committee of Qingdao Central Hospital, University of Health and Rehabilitation Sciences (Approval No. KY202606802). Given the retrospective nature of the analysis, the requirement for written informed consent was waived, as the study used anonymized data that did not compromise patient confidentiality. This study strictly adhered to the principles of the Declaration of Helsinki.

### 2.2 Patient selection

Patients were retrospectively identified if they had undergone AngioJet rheolytic thrombectomy using an 8-Fr system (Boston Scientific) with retrievable SVC filter protection for acute UEDVT between January 1, 2020, and October 1, 2025. Eligibility was determined at the discretion of the operator. The main inclusion criteria were symptomatic proximal UEDVT with a symptom duration of ≤14 days and an expected life expectancy of >6 months. Exclusion criteria were severe contrast allergy, renal insufficiency (defined as estimated glomerular filtration rate <30 mL/min/1.73 m^2^), and contraindication to anticoagulation. In this study, sex refers to biological sex (male or female) as recorded in the medical records.

### 2.3 Outcome indicators

#### 2.3.1 Primary outcome

The primary outcome was technical success rate defined as a thrombus clearance rate >80% as shown by postoperative venography. This evaluation was independently conducted by experienced vascular surgeons based on preoperative and postoperative imaging data.

#### 2.3.2 Secondary outcomes

The secondary outcomes included degree of relief of upper limb swelling (measured 10 cm proximal to the ulnar olecranon), bleeding events during the perioperative and follow-up periods, incidence of symptomatic and imaging-confirmed PE, and all-cause mortality and deep vein thrombosis recurrence rate during follow-up.

#### 2.3.3 Follow-up

The follow-up period extended from the day of surgery to the last follow-up date. Patients underwent regular assessments during the first 6 months after surgery, including examinations of limb symptoms and signs, venous ultrasound re-examinations, and recording of complications related to anticoagulation therapy.

### 2.4 Surgical procedure and postoperative management

All surgeries were performed under conscious sedation.

#### 2.4.1 Access establishment and filter placement

Access was established via puncture of the bilateral femoral veins. A retrievable conical SVC filter (LifeTech Scientific) was placed in the SVC via one femoral vein. After deployment of the SVC filter, the pre-existing central venous catheters were removed.

#### 2.4.2 Thrombus clearance

An AngioJet catheter was introduced via the contralateral femoral vein (or, if femoral access failed, via puncturing the ipsilateral brachial vein under ultrasound guidance) for PMT. The standard operating procedure involved first using the drug-spraying mode and then switching to the thrombus suction mode until effective thrombus clearance was achieved.

#### 2.4.3 Venous reconstruction

If there was >50% residual stenosis in the target venous segment, balloon venoplasty was performed. If stenosis persisted after balloon dilation, a venous stent was placed.

#### 2.4.4 Filter retrieval

At the end of the procedure, the filter was retrieved into the delivery sheath and withdrawn. Images from a representative example case are shown in the Figure.

**Figure:**
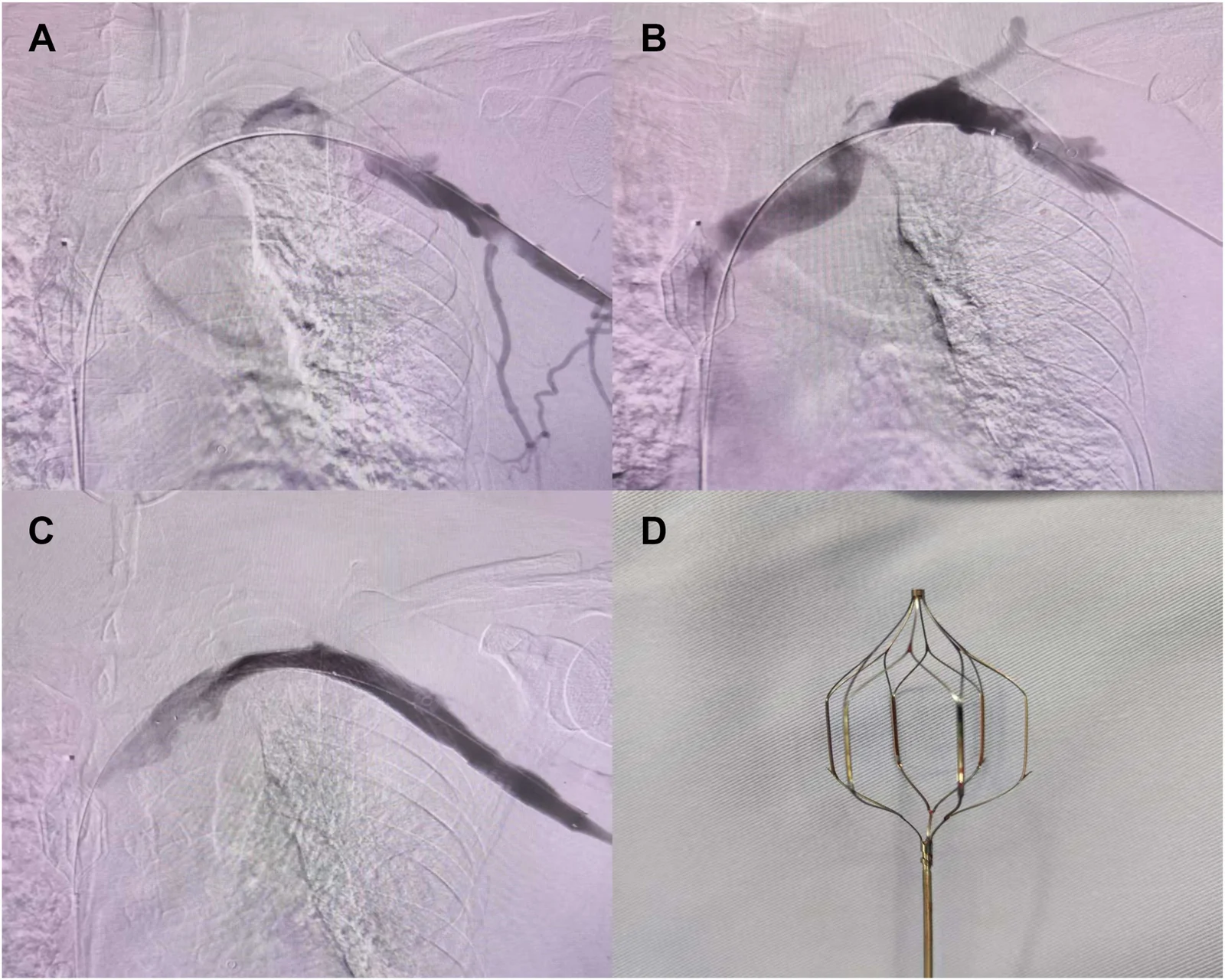
Mechanical thrombectomy and venous stent placement in a 52-year-old patient presenting with a 2-day history of left upper extremity pain and swelling. (A) Venography shows extensive thrombosis involving the subclavian, brachial, and axillary veins. (B) Post-thrombectomy venography shows a focal, severe stenosis in the subclavian vein. (C) Venography after stent placement showing restored venous flow patency. (D) A retrievable conical superior vena cava filter (LifeTech Scientific) used during the procedure.

#### 2.4.5 Postoperative management

Throughout the procedure, all patients were treated with therapeutic doses of either low-molecular-weight heparin or unfractionated heparin. After discharge, anticoagulation was continued with rivaroxaban. The standard regimen consisted of rivaroxaban 15 mg twice daily for the first 3 weeks, followed by 20 mg once daily thereafter. A reduced dose (rivaroxaban 15 mg or 10 mg once daily) was used selectively in patients with increased bleeding risk or moderate renal impairment, with the decision individualized according to renal function, bleeding risk, and concomitant medications. Anticoagulation was continued for at least 6 months, and extended to at least 1 year in patients who underwent stent placement.

### 2.5 Statistical analysis

Continuous variables are presented as mean ± standard deviation, and categorical variables are described using frequency (percentage). A paired *t*-test was used to compare limb circumferences before and after treatment. All statistical analyses were performed using SPSS (version 25.0; IBM).

## 3 Results

### 3.1 Patient baseline characteristics

A total of 19 patients (10 female; mean age 60.84 ± 11.30 years) with acute UEDVT were included. All patients had secondary UEDVT and a history of malignant tumors. The duration of preoperative limb swelling symptoms was ≤14 days for all. Among them, 14 patients (73.7%) had a history of central venous catheterization, and 13 patients (68.4%) had the affected limb on the left side. The average circumference of the affected limb 10 cm above the elbow before surgery was 29.83 ± 2.20 cm, which decreased to 26.98 ± 2.0 cm on the second postoperative day (*P* < .05). Imaging evaluations showed that all patients had subclavian vein thrombosis, with thrombus extending to the brachiocephalic vein in 11 patients (57.9%). Four patients (21.1%) had asymptomatic PE indicated by computed tomography pulmonary angiography before surgery. All patients underwent treatment within 2 days after imaging-confirmed diagnosis. Baseline characteristics of the patients included in the study are summarized in the Table.

**Table:** Baseline and procedural characteristics of all patients.

Characteristics	Value
Baseline features	
Age (y), mean ± SD	60.84 ± 11.30
Sex, *n* (%)	
Male	9 (47.4)
Female	10 (52.3)
Medical history, *n* (%)	
Malignancy	19 (100)
UEDVT catheterization	14 (74)
Diagnosis	
Secondary UEDVT, *n* (%)	19 (100)
Thrombus location, *n* (%)	
Brachiocephalic	11 (57.9)
Subclavian	19 (100)
Axillary	16 (84.2)
Brachial	11 (57.9)
jugular	5 (26.3)
Circumference (10 cm above elbow), mean ± SD	
Preoperative (cm)	29.83 ± 2.20
Postoperative day 2 (cm)	26.98 ± 2.0
Laterality, *n* (%)	
Right	6 (31.6)
Left	13 (68.4)
Procedural characteristics, *n* (%)	
Technical success	19 (100)
Vascular access	
Femoral and brachial	2 (10.5)
Femora	17 (89.5)
Balloon	16 (84.2)
Stent	9 (50)
Complications	
Major bleeding	0
Hematoma	0
Symptomatic PE	0
Transient bradycardia	5 (26.3)
Follow-up (6 mo)	
Symptomatic recurrence	1 (5.9)
Asymptomatic recurrence	1 (5.9)
Symptomatic PE	0
Death	2

PE, pulmonary embolism; UEDVT, upper extremity deep vein thrombosis.

### 3.2 Surgery and immediate outcomes

The technical success rate of the surgery was 100%, with all patients achieving a target venous thrombus clearance rate of >80%. All filters were successfully placed in the SVC via the femoral vein, with 2 patients switching to brachial access due to failure in establishing PMT access via the femoral vein. Nine patients (47.4%) underwent venous stenting for significant venous stenosis or occlusion, receiving a mean of 2 self-expanding venous stents (range, 1-4) per patient, with diameters of 10 to 14 mm (eg, Venovo [Bard/BD] or Wallstent [Boston Scientific]). Five patients experienced bradycardia during the procedure, which resolved immediately after stopping the device. At the time of retrieval, intrafilter thrombus was present in 4 of 19 filters (21.1%). The affected filters were carefully retracted into the delivery sheath, and no acute embolization occurred. No severe adverse events such as major bleeding occurred, nor were there any new symptomatic PE cases.

### 3.3 Follow-up results

During a median follow-up period of 6 months, 2 patients died of advanced malignancy at 130 days and 155 days after surgery, respectively. Apart from one patient who discontinued anticoagulation due to gastrointestinal bleeding 60 days after the procedure, all other patients adhered to the prescribed anticoagulation regimen. No symptomatic PE developed during the follow-up period. During the follow-up period, one patient with advanced lung cancer developed swelling of the upper limb on postoperative day 136, which was confirmed by ultrasound to be a recurrence of deep vein thrombosis; this patient chose conservative treatment. Another patient was found to have an asymptomatic mural thrombus on ultrasound examination on postoperative day 28, and under continuous anticoagulation (rivaroxaban) treatment, the limb showed no swelling at the outpatient follow-up on postoperative day 140.

## 4 Discussion

This single-center, retrospective study evaluated the clinical application of PMT combined with SVC filter protection in cancer patients with acute UEDVT. Our findings indicate a high technical success rate, satisfactory thrombus clearance, and no major procedural complications or symptomatic PE, suggesting that this combined strategy appears to be effective and safe. These results provide preliminary evidence supporting the use of PMT for acute UEDVT and may inform future optimization of treatment protocols aimed at reducing the risk of thrombus dislodgement.

Regarding the safety and efficacy of mechanical thrombectomy, previous studies have reported good safety and efficacy using the 6-Fr AngioJet system for treating UEDVT, showing positive outcomes [12]. This study is the first to systematically evaluate the clinical performance of the 8-Fr AngioJet system in acute UEDVT, with results showing no vascular rupture or severe bleeding events, and a technical success rate of up to 100%. This finding suggests that a larger diameter AngioJet catheter may provide better thrombus clearance efficiency, and its Bernoulli principle–based fluid dynamics system can generate optimized local negative pressure suction and thrombus fragmentation effects, thereby helping to reduce thrombus residue [[13], [14]]. In addition, the AngioJet system, based on the Bernoulli principle, may induce less traction injury to vascular endothelium, thus lowering the risk of mechanical damage to the vascular endothelium. By quantitatively measuring changes in limb circumference, the effectiveness of mechanical thrombectomy in restoring venous blood flow in the limbs was objectively assessed. Notably, the transient decrease in heart rate observed during the procedure reflected the occurrence of a vagal nerve reflex, the mechanism of which may be related to mechanical stimulation of the catheter within the vein, local pressure changes caused by suction, and the release of hemolytic products. These factors together lead to an imbalance between sympathetic and parasympathetic nerves, indicating that hemodynamics must be closely monitored during the procedure, with preparations in place to address severe bradycardia and hypotension [15].

The risk of PE due to thrombus migration is a potential risk of mechanical thrombectomy treatment for deep vein thrombosis, as emphasized in the 2025 European Society of Vascular Medicine guidelines [16]. Whether retrievable SVC filters should be used routinely or selectively during mechanical thrombectomy for UEDVT remains debated. Evidence is limited, and guidelines do not clearly favor either approach [17]. Routine filter protection may reduce the risk of clinically significant PE, especially in patients with extensive thrombus; however, selective placement minimizes complications related to filter insertion and retrieval, such as access-site thrombosis, filter migration or tilting, strut fracture, and rarely SVC perforation or cardiac tamponade [17]. This study combined an SVC filter protection strategy, successfully avoiding filter-related complications and PE events; the innovative operation of immediate removal of the filter at the end of the surgery significantly reduced the risk of device-related vascular injury, appearing a feasible new path for mechanical thrombectomy combined with filter protection. Given the lack of comparative data, the true safety benefit of routine temporary SVC filter protection requires evaluation in larger, ideally prospective, comparative studies.

Beyond technical success and venographic patency, treatment evaluation for UEDVT is increasingly emphasizing patient-centered outcomes such as symptom relief, functional recovery, and health-related quality of life. Although our study focused mainly on procedural and short-term clinical endpoints, we acknowledge that patient-reported outcomes were not systematically assessed. As highlighted by a recent multinational study by Abrishami Kashani et al. [18], even after successful intervention, patients may still experience persistent symptoms and functional limitations that imaging alone cannot fully capture.

Regarding anticoagulant therapy, all patients received direct oral anticoagulant therapy administered postoperatively for a minimum of 6 months. Deceased patients and one patient who temporarily discontinued therapy due to gastrointestinal bleeding were exceptions. Apart from these cases, the incidence of thrombotic recurrence was low. This result indicates that continuous anticoagulant therapy plays a crucial role in blocking the cytokine-mediated pathways of thrombus formation and inhibiting platelet activation [19]. Anticoagulants inhibit factor Xa and thrombin activity, reducing the formation of fibrin networks, thereby preventing further thrombus extension [20]. The follow-up data from this study support the effectiveness of anticoagulant therapy in preventing thrombus recurrence, consistent with previous efficacy of anticoagulation regimens for lower limb deep vein thrombosis; however, in the case of upper limb deep vein thrombosis, the specific duration and choice of medication still need to be individually adjusted based on the patient’s bleeding risk [21]. In patients with cancer, several unmeasured factors may confound thrombotic outcomes. Cancer-related vomiting may impair the oral bioavailability of direct oral anticoagulants, and potential drug-drug interactions with chemotherapeutic or supportive agents (eg, azole antifungals and certain tyrosine kinase inhibitors) could alter anticoagulant levels [22]. The lack of systematic documentation of these factors represents an important limitation of this study, as does the absence of systematic assessment of tumor stage, ongoing chemotherapy, and Eastern Cooperative Oncology Group performance status, which precluded determination of the optimal anticoagulation duration according to cancer activity or active oncological treatment. Furthermore, the presence of recurrent patients suggests that the mechanism of thrombus formation is complex and may involve resistance to anticoagulants or abnormalities in vascular endothelial function, and future research should focus on optimizing individualized strategies for anticoagulant therapy.

The main limitation of this study is the small sample size and its retrospective, single-center design, without a control group. The limited sample size may affect statistical power, and the lack of a control group makes it difficult to accurately assess the efficacy and safety of this technique, also limiting the generalizability of the conclusions. Chronic venous symptoms such as post-thrombotic syndrome can develop or progress months to years after the index event, and the true rates of recurrent thrombosis and the durability of functional recovery can only be assessed with extended observation. Future multicenter, large-scale prospective studies are needed for further validation.

In conclusion, PMT using the 8-Fr AngioJet system with SVC filter protection appears to be a safe and effective treatment option for cancer patients with acute UEDVT. Although the use of an SVC filter may mitigate the risk of intraoperative PE, prospective studies are still needed to confirm these findings and establish optimal management strategies for UEDVT.
